# Elucidation of Relevant Neuroinflammation Mechanisms Using Gene Expression Profiling in Patients with Amyotrophic Lateral Sclerosis

**DOI:** 10.1371/journal.pone.0165290

**Published:** 2016-11-03

**Authors:** Yu Hui Won, Min-Young Lee, Young-Chul Choi, Yoon Ha, Hyongbum Kim, Do-Young Kim, Myung-Sun Kim, Ji Hea Yu, Jung Hwa Seo, MinGi Kim, Sung-Rae Cho, Seong-Woong Kang

**Affiliations:** 1 Department of Physical Medicine and Rehabilitation, Research Institute of Clinical Medicine of Chonbuk National University-Biomedical Research Institute of Chonbuk National University Hospital, Jeonju, Korea; 2 Department and Research Institute of Rehabilitation Medicine, Yonsei University College of Medicine, Seoul, Korea; 3 Department of Neurology, Gangnam Severance Hospital, Yonsei University College of Medicine, Seoul, Korea; 4 Department of Neurosurgery, Spine & Spinal Cord Institute, College of Medicine, Yonsei University, Seoul, Korea; 5 Department of Pharmacology, Yonsei University College of Medicine, Seoul, Korea; 6 Department of Dermatology, Cutaneous Biology Research Institute, Yonsei University College of Medicine, Seoul, Korea; 7 Brain Korea 21 PLUS Project for Medical Science, Yonsei University, Seoul, Korea; 8 Department of Rehabilitation Medicine, Gangnam Severance Hospital, Rehabilitation Institute of Neuromuscular Disease, Yonsei University College of Medicine, Seoul, Korea; 9 Department of Medicine, the Graduate School of Yonsei University, Seoul, Korea; CHA University, REPUBLIC OF KOREA

## Abstract

Amyotrophic lateral sclerosis (ALS) is a progressive neurodegenerative disorder characterized by damage of motor neurons. Recent reports indicate that inflammatory responses occurring within the central nervous system contribute to the pathogenesis of ALS. We aimed to investigate disease-specific gene expression associated with neuroinflammation by conducting transcriptome analysis on fibroblasts from three patients with sporadic ALS and three normal controls. Several pathways were found to be upregulated in patients with ALS, among which the toll-like receptor (TLR) and NOD-like receptor (NLR) signaling pathways are related to the immune response. Genes—toll-interacting protein (*TOLLIP*), mitogen-activated protein kinase 9 (*MAPK9*), interleukin-1β (*IL-1β*), interleukin-8 (*IL-8*), and chemokine (C-X-C motif) ligand 1 (*CXCL1*)—related to these two pathways were validated using western blotting. This study validated the genes that are associated with TLR and NLR signaling pathways from different types of patient-derived cells. Not only fibroblasts but also induced pluripotent stem cells (iPSCs) and neural rosettes from the same origins showed similar expression patterns. Furthermore, expression of TOLLIP, a regulator of TLR signaling pathway, decreased with cellular aging as judged by changes in its expression through multiple passages. TOLLIP expression was downregulated in ALS cells under conditions of inflammation induced by lipopolysaccharide. Our data suggest that the TLR and NLR signaling pathways are involved in pathological innate immunity and neuroinflammation associated with ALS and that TOLLIP, MAPK9, IL-1β, IL-8, and CXCL1 play a role in ALS-specific immune responses. Moreover, changes of TOLLIP expression might be associated with progression of ALS.

## Introduction

Amyotrophic lateral sclerosis (ALS) is a fatal neurological disease characterized by damage of motor neurons, microglial activation, and wide astrogliosis in the motor cortex and spinal cord [1, 2]. Several genes associated with the disease have been identified; however, the pathogenic mechanisms are still elusive, and so far no curative therapeutic treatment has been developed. Suggested pathogenic mechanisms of ALS contain genetic factors, glutamate excitotoxicity, oxidative stress, weakened axonal transport, changed protein turnover, apoptosis, mitochondrial dysfunction, neurotrophic deficiency, and neuroinflammation [3]. Evidence accumulated over the past decade indicates that immune activation and inflammation might be implicated in ALS pathogenesis. Astrocytes, microglia, and immune-related cells have all been shown to be actively involved in ALS pathogenesis [2, 4–8]. The immune responses associated with neurodegenerative changes have been termed neuroinflammation. In pathologically affected areas of the central nervous system (CNS) in both human patients with ALS and mouse models of the disease, noticeable neuroinflammation can be observed [2].

With these lines of evidence indicating that neuroinflammation is related to the pathogenesis of ALS, we hypothesized that mRNA expression of components of immune-related signaling pathways would be different in patients with sporadic ALS and that such differences might reveal the pathogenic factors related to neuroinflammation. To identify differentially expressed genes involved in the pathogenesis of ALS, we carried out gene expression profiling of fibroblast cells from patients with ALS and normal controls using RNA sequencing transcriptome analysis. In addition, we studied the certain inflammation-related genes in three different types of cells—fibroblasts, iPSCs, and neural lineage cells, and confirmed the genes that were considered to be representing certain inflammatory pathways. The aim of this study was to explore disease-specific gene expressions associated with neuroinflammation in ALS using gene expression profiling in order to better comprehend the pathogenesis of ALS.

## Materials and Methods

### Subjects

The study was approved by the Ethics Committee and the participants signed informed consent prior to the study. The human fibroblast samples were obtained with approval of participants with their written informed consent to participate in this study. The Institutional Review Board of Severance Hospital, Yonsei University Health System approved this consent procedure and the entire study (no. 4-2012-0028).

Patients with sporadic ALS who were diagnosed using El Escorial criteria [9] and normal healthy individuals free from any pharmacological treatment were included in this study. These three healthy individuals have been represented as normal controls in the following content. The patients were recruited at the Rehabilitation Institute of Neuromuscular Disease. All patients with ALS had been previously screened for *SOD1* gene mutation and showed no mutation. Regarded on personal health histories obtained by interviews, control donors were all unrelated and the normal phenotype was identified. To assess total functional condition of patients, the Revised ALS Functional Rating Scale (ALSFRS-R), scored 0–48, was used [10]. All scores were recorded within a week of dermal punch biopsy. None of the patients with ALS or normal subjects included in the study displayed signs of infection before biopsy.

### Preparation of fibroblast cells

Punch biopsy was conducted by a dermatology specialist and performed in the upper lateral quadrant of the buttock in patients with confirmed ALS. Dermal fibroblasts were also obtained from normal subjects. The biopsy sample was transferred to a culture dish in Dulbecco’s modified Eagle’s medium (DMEM) containing 10% fetal bovine serum (FBS) and penicillin/streptomycin and incubated in a humidified 5% CO_2_ atmosphere at 37°C.

### Cell proliferation and senescence analysis

For analysis of cell proliferation fibroblasts were seeded in 6-well plates (10,000 cells/plate) in DMEM containing 10% FBS and penicillin/streptomycin. The number of cells per plate was determined from counts obtained with an ADAM automatic cell counter 2, 4, 6, and 8 days after plating as described in the manufacturer’s protocol (NanoEnTek Inc, South Korea).

Flow cytometric analysis of cellular senescence was carried out using a Quantitative Cellular Senescence Assay Kit (Cell Biolabs, San Diego, CA, USA). Briefly, fibroblasts were treated with pretreatment solution at 37°C for 2 h. Next, senescence-associated β-galactosidase (SA-β-gal) substrate solution was added to the cells for 4 h. The stained cells were washed with phosphate-buffered saline (PBS), harvested by trypsinization, and flow cytometric analysis was performed in PBS containing 1% FBS on a FACSLSRII flow cytometer (BD Bioscience, San Jose, CA, USA). For microscopy studies, fibroblasts were washed with PBS, fixed for 15 min with the fixing solution at room temperature, briefly washed in PBS, and incubated with SA-β-Gal substrate solution at 37°C without CO_2_ and with protection from light for 16 h. The blue stained cells were analyzed under light microscopy.

### Generation of iPSCs

The following protocol was previously described [11]. Following to the protocol of manufacturer’s, episomal vector mixtures (total 3 μg) encoding defined reprogramming factors were electroporated by using a microporator system (Neon; Invitrogen, Carlsbad, CA, USA). After being pulsed three times with a voltage of 1,650 for 10 ms, the cells were grown further in DMEM (containing 10% FBS). Otherwise, CytoTune^TM^ Sendai virus solution (Thermo Fisher Scientific, Waltham, MA) including defined reprogramming four factors is mixed, and added onto ALS and normal fibroblasts (MOI = 3). Seven days after transfection or transduction, cells were transferred onto a feeder layer. iPSC colonies similar to human embryonic stem cells (hESCs) were picked up mechanically and further cultured for characterization.

### Cell cultures for iPSCs

Human iPSCs (normal and ALS) were cultured on mouse SIM Thioguanine/Ouabain-resistant mouse fibroblast cell line (STO) under previously described growth conditions [11]. Human iPSCs (normal and ALS) generated in this study were maintained in hESC medium composed of DMEM/F12 medium supplemented with 20% (vol/vol) knockout serum replacement (Invitrogen, Carlsbad, CA), 4.5 g/L L-glutamine, 1% nonessential amino acids, 0.1 mM 2-mercaptoethanol, and 10 ng/mL basic fibroblast growth factor (bFGF) (Invitrogen, Carlsbad, CA)

### Induction of neural rosettes

For induction of neural rosettes, Embryonic bodies (EBs) were cultured in suspension for 4 days in hESC media excluding bFGF but supplemented with 5mM dorsomorphin (DM) (Calbiochem, Darmstadt, Germany) and 5 to 10 μM SB431542 (SB) (Sigma, St. Louis, MO, USA). On day 4, EBs were attached in Matrigel-coated culture dishes (BD Biosciences, San Jose, CA, USA) in DMEM/F12 N2 supplemented media (N2 media) with 20 ng/ml bFGF (R&D Systems, Minneapolis, USA) and 19 to 21 μg/ml human insulin solution (Sigma, St. Louis, MO) for another 5 days. The emerged rosette structures were mechanically isolated using pulled glass pipettes within the EB colonies, and isolated neural rosette clumps were replaced in Matrigel-coated dishes. Replated neural rosettes were then expanded for an additional 6 to 7 days at 90% confluence [12].

### RNA preparation

According to the manufacturer’s instructions, total RNA was isolated from cultured fibroblasts obtained from patients with ALS and normal subjects using Trizol (Invitrogen Life Technologies, Carlsbad, CA, USA) [13]. RNA purity was evaluated with an Agilent 2100 Bioanalyzer (Agilent Technologies, Palo Alto, CA, USA) and measured by the A260/A280 ratio for quality control analysis. RNA integrity was evaluated by visual valuation of the 28S:18S rRNA ratio using gel electrophoresis.

### RNA sequencing and transcriptome data analysis

RNA sequencing was performed by Macrogen Inc (Seoul, Korea). The mRNA was changed into a library of templates suitable for successive cluster generation using the reagents provided in the Illumina^®^ TruSeq™ RNA Sample Preparation Kit [14–16]. The transcriptome analysis is composed of RNA-seq experiment and data handling. RNA-seq experiment is TruSeq mRNA library construction which is organized by 8 steps: purify and fragment mRNA, synthesize first strand cDNA, synthesize second strand cDNA, perform end repair, adenylate 3' ends A single, ligate adapters, enrich DNA fragments, and enriched library validation. First, using magnetic beads which poly-T oligo-attached, purifying the poly-A containing mRNA molecules. After purification, using divalent cations under raised temperature, the mRNA is split into small fragments. The cleaved RNA fragments primed with random hexamers into first strand cDNA using random primers and reverse transcriptase. These cDNA fragments are then subjected to an end repair process using an end repair (ERP) mix involving the addition of a single “A” nucleotide, followed by ligation of the adapters. On the 3’ end of the adapter, there is a corresponding single ‘T’ nucleotide, and it provides a paired overhang for binding the adapter to the fragment. To create the final cDNA library, the products are purified and enriched by PCR [17]. Illumina utilizes a unique reaction. The reaction is called “bridged” amplification reaction which reacts on the surface of the flow cell. A flow cell, which contains millions of unique clusters, is loaded into the HiSeq 2000 for automated cycles of imaging and extension. Solexa’s Sequencing-by-Synthesis utilizes four proprietary nucleotides with reversible fluorophore and termination properties. Every individual sequencing cycle occurs in the presence of all four nucleotides, leading to better accuracy than methods in which only one nucleotide is present in the reaction mix [16]. To evaluate expression levels, RNA-seq reads were mapped to the human genome. Transcript counts at the gene level were calculated. Data analysis performed by TopHat and Cufflinks. For RNA-seq reads, TopHat is a fast splice junction mapper. Using the ultra-high-throughput short read aligner Bowtie, TopHat aligns RNA-seq reads to mammalian-sized genomes. After then, for identifying the splice junctions between exons, it analyzes the mapping results. Cufflinks tests for differential expression and regulation in RNA-seq samples and assembles transcripts, estimates their abundances. Transcripts with a fold induction ≥2 and Benjamin-Hochberg adjusted *p* value ≤ 0.05 were considered significant and were included in downstream analysis.

### Pathway analysis

We used the database for annotation, visualization, and integrated discovery (DAVID) software (http://david.abcc.ncifcrf.gov/) [18] and Kyoto Encyclopedia of Genes and Genomes (KEGG) pathways [19, 20] for analysis. Using DAVID software, we searched for pathways that were upregulated in subjects with ALS. Within these identified pathways, genes that are also involved in neuroinflammation were selected for further validation.

### Reverse transcription polymerase chain reaction (RT-PCR)

To validate the results of transcriptome analysis, we performed RT-PCR of genes that were components of significantly upregulated KEGG pathways and known to be involved in neuroinflammation. For RT-PCR, the following reaction-specific primers were used: cluster of differentiation 14 (*CD14)*, forward 5′-cagcctagacctcagccaca-3′ and reverse 5′-tcccgtccagtgtcaggtta-3′; interferon-α/β receptor-1 (*IFNAR-1*), forward 5′-cgatgagtctgtcgggaatg-3′ and reverse 5′-gaccaatctgagctttgcga-3′; toll-interacting protein (*TOLLIP*), forward 5′-aagaatccccgctggaataa-3′ and reverse 5′-gaggttgatcatgccctcct-3′; phosphatidylinositol 4,5-bisphosphate 3-kinase catalytic subunit α (*PIK3CA*), forward 5′-attccagacgcatttccaca-3′ and reverse 5′-gagcagcacgaggaagatca-3′; interleukin-1 receptor-associated kinase 4 (*IRAK4*), forward 5′-agcttgcagcaatggttgac-3′ and reverse 5′-tagctgcaccctgagcaatc-3′; mitogen-activated protein kinase 1 (*MAPK1*), forward 5′-accaaccatcgagcaaatga-3′ and reverse 5′-acggtgcagaacgttagctg-3′; mitogen-activated protein kinase 9 (*MAPK9)*, forward 5′-cggacagcgtgcactaactt-3′ and reverse 5′-tttcaccagctctcccatga-3′; interleukin-1β (*IL-1β*), forward 5′-gtacctgagctcgccagtga-3′ and reverse 5′-tgaagcccttgctgtagtgg-3′; interleukin-8 (*IL-8)*, forward 5′-caaacctttccaccccaaat-3′ and reverse 5′-accctctgcacccagttttc-3′; chemokine (C-X-C motif) ligand 1 (*CXCL1*), forward 5′-tcaccccaagaacatccaaa-3′ and reverse 5′-actatgggggatgcaggatt-3′; caspase recruitment domain-containing protein 9 (*CARD9*), forward 5′-gatgtacaaggaccgcatcg-3′ and reverse 5′-gcctcacactggaacacctg-3′; glyceraldehyde-3-phosphate dehydrogenase (*GADPH)*, forward 5′-caaggtcatccatgacaactttg-3′ and reverse 5′-gtccaccaccctgttgctgtag-3′. The *GAPDH* gene was used as the internal control. Using RT-PCR, this study confirmed the induction of inflammation with the following targets. cyclooxygenase-2 (*COX-2*) forward 5′-cttcacgcatcagtttttcaag-3′ and reverse 5′-tcaccgtaaatatgatttaagtccac-3′. To confirm the iPSCs by RT-PCR, *OCT-3/4*, forward 5’-atcctgggggttctatttgg-3’ and reverse 5’-ctccaggttgcctctcactc-3’; *NANOG*, forward 5’-ttccttcctccatggatctg-3’ and reverse 5’-tctgctggaggctgaggtat-3’; SRY-box 2 (*SOX2*), forward 5’-aaccccaagatgcacaactc-3’ and reverse 5’-cggggccggtatttataatc-3’. To confirm the neural rosettes, *NESTIN*, forward 5’-gaaacagccatagagggcaaa-3’ and reverse 5’-tggttttccagagtcttcagtga-3’; paired box 6 protein (*PAX6*), forward 5’-gtgtccaacggatgtgtgag-3’ and reverse 5’-ctagccaggttgcgaagaac-3’; forkhead box G1 (*FOXG1*), forward 5’-aggagggcgagaagaagaac-3’ and reverse 5’-tcacgaagcacttgttgagg-3.

### Real time quantitative polymerase chain reaction (RT-qPCR)

As described in detail in the previous study [21], for additional validation, real time quantitative PCR (RT-qPCR) was performed in triplicate on a LightCycler 480 (Roche Applied Science, Mannheim, Germany) using the LightCycler 480 SYBR Green master mix (Roche Applied Science), and thermocycler conditions of 10 min template preincubation step at 95°C followed by 40 cycles at 95°C for 10 s, 60°C for 10 s, and 72°C for 10 s. The melting curve analysis began at 95°C for 5 s, followed by 1 min at 60°C. The specificity of the produced amplification product was confirmed by the melting curve analysis and showed a distinct single sharp peak with the expected *T*m for all samples. A distinct single peak indicates that a single DNA sequence was amplified during RT-qPCR. The *GAPDH* gene was used as the internal control. The expression level of each gene of interest was obtained using the 2^*−ΔΔCt*^ method.

### Western blotting

As mentioned in the previous study [21], to confirm the expression of genes that were validated by RT-PCR and RT-qPCR, 30 μg of extracted proteins were dissolved in sample buffer, boiled for 5 min, and loaded onto a 10% sodium dodecyl sulfate (SDS) reducing gel. The separated proteins were then blotted onto polyvinylidene difluoride membranes (Amersham Pharmacia Biotech, Little Chalfont, UK) using a transblot system (Bio-Rad, Hercules, CA, USA) at 100V for 1 h. The membranes were blocked for 1 h in Tris-buffered saline (TBS) containing 5% nonfat dry milk (Bio-Rad), washed three times with TBS containing 0.01% Tween 20 (TBST) for 15 min, and incubated overnight at 4°C with first antibodies specific to the target proteins. The first antibodies are TOLLIP (1:1000 Abcam, Cambrige, MA), MAPK9 (1:1,000 Abcam, Cambrige, MA), IL-1β (1:1,000 Abcam, Cambrige, MA), IL-8 (1:1,000 Abcam, Cambrige, MA), CXCL1 (1:1,000 Abcam, Cambrige, MA), and GAPDH (1:1,000 Santa Cruz Biotechnology, CA, USA). The next day, the blots were washed three times with TBST and incubated for 1 h with horseradish peroxidase (HRP)-conjugated secondary antibodies (1:3,000 Santa Cruz Biotechnology, CA). After washing the blots three times with TBST, the blots were visualized with an enhanced chemiluminescence detection system (Amersham Pharmacia Biotech) [22].

### Alkaline phosphatase staining and immunostaining

Alkaline phosphatase (AP) activity was measured with the leukocyte AP staining kit (System Biosciences, CA, USA) according to the manufacturer’s instructions. For detecting AP activity, the 4% paraformaldehyde fixed cells were stained AP solution for 20 min in room temperature (RT). Samples were observed with inverted microscope (NIKON, Japan). For immunostaining of pluripotent stem cell markers such as OCT4 and SSEA4, cells were fixed in 4% paraformaldehyde solution (20min, RT), permeabilized with 0.1% Triton X-100 (10min, RT), and blocked in 10% goat serum (1 h, RT). The iPSCs were incubated with primary antibodies such as OCT3/4 (Santa Cruz, Texas, USA) and SSEA4 (Stemgent, MA, USA). The cells were observed under fluorescent microscope (Cal Zeiss, Germany).

### Lipopolysaccharide (LPS) and IL-1β treatment

Normal and ALS fibroblast cells were seeded in 6-well plates and maintained until they reached 80% confluence before incubation in DMEM serum-free media (SFM). Fibroblast cells were washed five times with PBS and then treated with or without stimulation with 10 ng/mL LPS (Sigma, St. Louis, MO) for 3 h. To confirm the induction of inflammation, expression of human IL-1β was validated using RT-PCR. Expression of TOLLIP was validated by RT-PCR of the cultured cells after LPS treatment at passages 4, 8, and 12. Normal- and patient-derived iPSCs were washed five times with PBS and then treated with 1 μg/mL or 5 μg/mL LPS for 24 h, or remained without any treatment. Normal- and patient-derived iPSCs and neural rosettes were washed five times with PBS and then treated with 10 ng/mL or 100 ng/mL hIL-1β (R&D Systems, Minneapolis) for 3 h, or remained without any treatment. To confirm the induction of inflammation, expression of COX-2 was validated using RT-PCR. Expression of TOLLIP was validated by RT-PCR of the cultured cells after hIL-1β treatment.

### Sequencing analysis

Genomic DNA extracted from this individual’s sample was used for library preparation. Massively parallel sequencing was done on the MiSeq System (Illumina). Additionally the primers C9ORF72 forward 5'-ccagcttcggtcagagaagaaat-3’ and reverse 5'-gggtctagcaagagcaggtg-3’ were used for the PCR. The PCR reaction was performed with 20 ng of genomic DNA as the template in a 30 μℓ reaction mixture by using a *EF-Taq* (SolGent, Korea) as follows: activation of *Taq* polymerase at 95°C for 5 min, 35 cycles of 95°C for 1 min, 58°C, and 72°C for 1 min each were performed, finishing with 10 min step at 72°C. The amplification products were purified with a multiscreen filter plate (Millipore Corp., Bedford, MA, USA). Sequencing reaction was performed using a PRISM BigDye Terminator v3.1 Cycle sequencing Kit. The DNA samples containing the extension products were added to Hi-Di formamide (Applied Biosystems, Foster City, CA, USA). The mixture was incubated at 95°C for 5 min, followed by 5 min on ice and then analyzed by ABI Prism 3730XL DNA analyzer (Applied Biosystems, Foster City, CA).

### Statistical analysis

Data were expressed as mean ± standard deviation. Using Statistical Package for Social Sciences (SPSS) version 20.0, statistical analyses were performed. Non-parametric statistical analysis such as Mann-Whitney U test was used for the comparison of two groups. Student *t*-test was also used to confirm the statistical results. A *P-*value <0.05 was considered statistically significant.

## Results

### Characteristics of subjects

Dermal punch biopsies were taken from three patients with ALS and three normal controls to obtain fibroblast cells for this study. Basic demographic and clinical information of the study subjects are described in Table 1. Mean disease duration of ALS was 27.7 months. Two patients with ALS were managed with percutaneous endoscopic gastrostomy (PEG) and overnight noninvasive ventilator, whereas subject 3 showed relatively mild symptomatic manifestations of dysarthria and dysphagia. In the patient number 3 case, as the disease progression had been rapidly worse, six months after the skin biopsy, the patient experienced respiratory dysfunction and dysphagia. Thus, since then, the patient number 3 also had used ventilator and PEG tube like the same as patient number 1 and 2 (Table 1).

**Table 1:** Characteristics of patients with amyotrophic lateral sclerosis (ALS).

	Subject 1	Subject 2	Subject 3
**Age**	45	51	67
**Male / Female**	Male	Female	Female
**Disease duration (months)**	38	33	12
**ALSFRS-R**	27	27	46
**Ventilator use**	Overnight NIV use	Overnight NIV use	None
**Feeding**	PEG tube	PEG tube	Oral feeding

VC, vital capacity; PEG, percutaneous endoscopic gastrostomy; NIV, noninvasive ventilation

Normal controls were all males, aged 32, 63, and 76 years respectively. Mean ages of patients with ALS and normal controls were 54.3 years and 57.0 years, respectively. Performing genetic analysis on three ALS cases with no shared genetic similarities (at least we are not told whether they are from a specific mutation or family) is irrelevant. To verify the above, we selected certain genes that have been reported as ALS-related genes, and confirmed the sequence using genomic DNA (gDNA) from patient-derived fibroblasts. When massively parallel sequencing was done on the MiSeq System (Illumina) to verify mutant genes related with ALS, there were no ALS-related mutant genes including SOD1, TDP-43, FUS, UBQLN2 and VCP that cause disease.

Furthermore, we confirmed the GGGGCC (G_4_C_2_) hexanucleotide repeat expansion in *C9orf72* which affects ALS pathogenic phenomenon. The diagnosis of *C9orf72*-related ALS/FTD is established by detection of a heterozygous pathogenic GGGGCC (G_4_C_2_) hexanucleotide repeat expansion in *C9orf72* on molecular genetic testing [23–26]. When this study confirmed the sequence of gDNA from the fibroblasts to count the exact repeat numbers of GGGGCC (G_4_C_2_) hexanucleotide in C9ORF72, there were two patients with double repeat of GGGGCC (G_4_C_2_), and another did not have any GGGCC (G_4_C_2_) hexanucleotide repeat (S1 Fig). Because this GGGCC (G_4_C_2_) hexanucleotide repeat number is not the significant repeat to show pathogenic phenomena, we concluded that these ALS patients were not genetic but sporadic.

### Cell proliferation and cellular senescence

**Fig 1:**
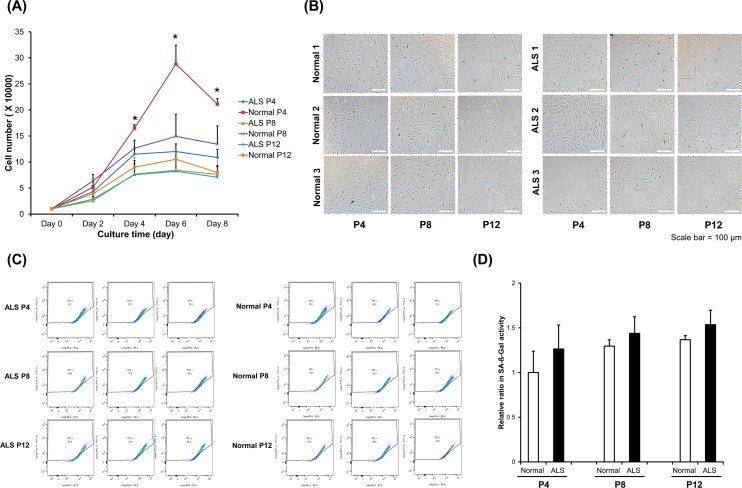
Cellular proliferation and senescence. (A) Growth curves of fibroblasts from patients with ALS and normal subjects at passages 4, 8, and 12 during culture for 8 days (*P < 0.05). (B) β-galactosidase (SA-β-Gal) activity of fibroblasts from patients 1, 2, and 3 with ALS and normal subjects 1, 2, and 3 at passages 4, 8, and 12. This image magnified by a factor of 100 (Scale bar, 100 μm). (C) Flow cytometric analysis of fibroblasts from patients with ALS and normal subjects at passages 4, 8, and 12. (D) A graph showing the relative ratio of SA-β-Gal activity on normal subjects and patients with ALS at passages 4, 8, and 12.

To evaluate cell proliferation, fibroblasts from 6-well plates were harvested and counted with an automatic cell counter on days 2, 4, 6, and 8. In addition, the growth curves of cultured fibroblasts at passages 4, 8, and 12 were generated. When cell proliferation was observed until passages 12, fibroblasts from normal subjects in passages 4 exhibited a significantly higher proliferation rate than fibroblasts from patients with ALS, which showed a slow growth rate (*p*<0.05). The proliferation of fibroblasts from both normal subjects and patients with ALS decreased after passages 8 (Fig 1A).

To evaluate cellular senescence, the senescence status of fibroblasts from normal subjects and patients with ALS was investigated by performing morphometric analysis for SA-β-Gal activity. When fibroblasts from patients with ALS were cultured in passages 4, the cells had already entered a senescent state compared to fibroblasts from normal subjects. Fibroblasts from patients with ALS displayed more positive SA-β-Gal staining by light microscopy (Fig 1B). When flow cytometric analysis was concurrently performed to quantify the SA-β-Gal activity, fibroblasts from both normal subjects and patients with ALS showed an increase in SA-β-Gal activity with progression of passages (Fig 1C). Fibroblasts from patients with ALS showed a higher level of senescence than fibroblasts from normal subjects, as indicated by relative ratio for SA-β-Gal activity (Fig 1D). Even though there was no statistical significance, senescence showed an increasing pattern while the cell passages were increasing.

### Differentially expressed genes and pathway analysis

RNA was prepared from cultured fibroblasts of patients with ALS and normal subjects at passages 4. We performed transcriptome analysis by RNA sequencing to identify genes that were differentially expressed in patients with ALS compared to normal controls; Total 17,025 genes were differentially expressed, and listed (S1 Table). Among total genes at the ALS patients, 626 transcripts were 2.0-fold lower, and 589 transcripts were higher than normal control samples (S2 Table).

Using DAVID software, several pathways were identified to be significantly upregulated in subjects with ALS (Table 2). Among these pathways, the Toll-like receptor (TLR) signaling pathway and NOD-like receptor (NLR) signaling pathway are related to innate immunity, and these two pathways are statistically significant (*p*<0.05). Significantly upregulated genes related to TLR signaling pathway were IRAK4, MAPK1, IL-8, TOLLIP, IL-1β, MAPK9, PIK3CA, CD14, and IFNAR-1. In addition, genes related to the NLR signaling pathway such as CXCL1, MAPK1, CARD9, IL-8, IL-1β, and MAPK9 were significantly upregulated.

**Table 2:** Kyoto Encyclopedia of Genes and Genomes (KEGG) pathways identified to be significantly upregulated from the fibroblasts of patients with ALS using Database for Annotation, Visualization and Integrated Discovery (DAVID) software.

KEGG pathway	Count	*P*-value	Genes
Axon guidance	11	0.0049*	MAPK1, NCK2, HRAS, PLXNC1, RGS3, FYN, CFL2, EFNB2, PPP3CB, SEMA4B, SEMA3B
Cell cycle	10	0.012*	ANAPC1, FZR1, CDKN2A, HDAC2, PLK1, CDC20, PTTG1, CDC26, CDC27, TFDP1
Ubiquitin mediated proteolysis	10	0.0207*	ANAPC1, FZR1, UBE4A, PML, UBE2W, CDC20, UBE2J2, CDC26, UBE2C, CDC27
Toll-like receptor signaling pathway	9	0.0102*	IRAK4, MAPK1, IL8, TOLLIP, IL1B, MAPK9, PIK3CA, CD14, IFNAR1
NOD-like receptor signaling pathway	6	0.0369*	CXCL1, MAPK1, CARD9, IL8, IL1B, MAPK9

These pathways are statistically significant (*P < 0.05).

### Validation of expression results by RT-PCR, RT-qPCR and western blotting

**Fig 2:**
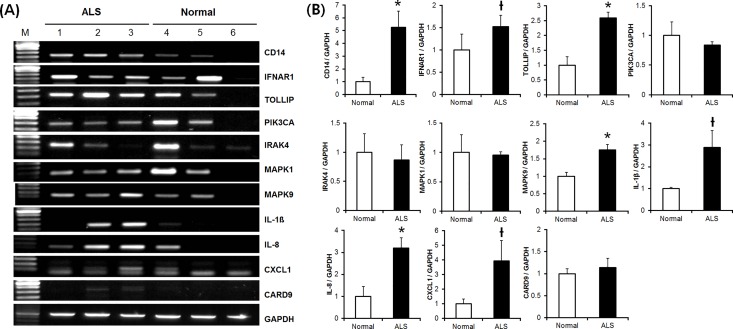
RT-PCR verification of genes identified in transcriptome analysis. (A) RT-PCR analysis of 11 genes. (B) Comparison of relative gene expression in normal subjects and patients with ALS. Eleven genes from the Toll-like receptor and NOD-like receptor signaling pathways were verified using RT-PCR and genes—*CD14*, interferon-α/β receptor-1 (*IFNAR-1*), toll-interacting protein (*TOLLIP*), mitogen-activated protein kinase 9 (*MAPK9*), interleukin 1 beta (*IL-1β*), interleukin 8 (*IL-8*), and chemokine (C-X-C motif) ligand 1 (*CXCL1*)—were upregulated in the ALS group. *P < 0.05, ^†^*P* < 0.1

We next performed RT-PCR of the above 11 genes for validation. Expression levels of these genes in ALS patient-derived fibroblasts were relative in fibroblasts from normal subjects. This result was determined by RT-PCR as follows: *CD14* (5.25-fold, *p* = 0.024), *IFNAR-1* (1.53-fold, *p* = 0.094), *TOLLIP* (2.59-fold, *p*<0.001), *PIK3CA* (0.84-fold, *p* = 0.436), *IRAK4* (0.87-fold, *p* = 0.730), *MAPK1* (0.96-fold, *p* = 0.863), *MAPK9* (1.76-fold, *p*<0.001), *IL-1β* (2.89-fold, *p* = 0.077), *IL-8* (3.20-fold, *p* = 0.004), *CXCL1* (3.92-fold, *p* = 0.063), and *CARD9* (1.14-fold, *p* = 0.387) (Fig 2A and 2B).

**Fig 3:**
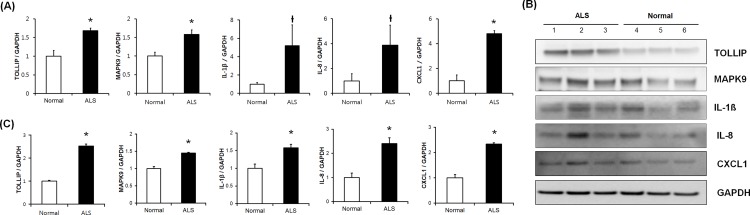
Validation of identified genes by RT-qPCR and western blotting. (A) Quantitative comparison of relative gene expressions between normal subjects and ALS patients by RT-qPCR. (B) TOLLIP, IL-1β, IL-8, MAPK9, and CXCL1 proteins were overexpressed in the subjects with ALS by western blotting. (C) Comparison of relative expression from normal subjects and patients with ALS for the five proteins verified by western blotting. *P < 0.05, ^†^*P* < 0.1

We performed RT-qPCR for more accurate quantitative analysis. As a result, we confirmed the similar expression patterns like the result of RT-PCR (Fig 3A). This result was determined by RT-qPCR as follows: *TOLLIP* (1.67-fold, *p* = 0.002), *MAPK9* (1.57-fold, *p* = 0.004), *IL-1β* (5.20-fold, *p* = 0.069), *IL-8* (3.86-fold, *p* = 0.099), and *CXCL1* (4.8-fold, *p* = 0.041).

Western blotting was performed for further validation for the significantly upregulated genes that were confirmed by RT-PCR. The amount of protein encoded by these genes in fibroblasts from patients with ALS relative to that in fibroblasts from normal subjects as determined by western blotting was as follows: TOLLIP (2.55-fold, *p*<0.001), MAPK9 (1.45-fold, *p*<0.001), IL-1β (1.57-fold, *p* = 0.004), IL-8 (2.40-fold, *p*<0.001), and CXCL1 (2.33-fold, *p*<0.001) (Fig 3B and 3C).

### Comparison of differential gene expression between normal and ALS patient-derived iPSCs and neural rosettes

Since ALS is a CNS disease, there is a limitation on gene screening between fibroblasts cultured from normal controls and patients. To overcome the limitation, we established iPSCs derived from fibroblasts and generated neural rosettes differentiated from the iPSCs. When the expression patterns of inflammation-related genes in three different types of cells were compared between normal controls and ALS patients, these different types of cells showed similar gene expression patterns.

**Fig 4:**
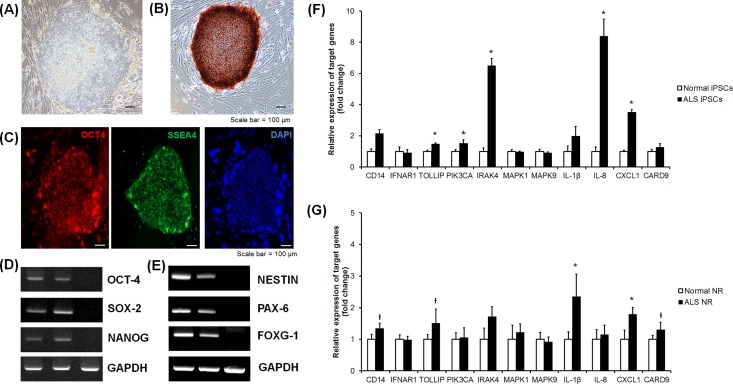
Verification of genes identified in transcriptome analysis in normal- and patient-derived iPSCs and neural rosettes. (A) Morphology of the expanded human iPSCs (Scale bar, 100 μm). (B) Alkaline phosphatase staining of iPSCs (Scale bar, 100 μm). (C) The expression of *OCT4* and *SSEA4*, which are human ESC-specific markers, was detected by immunocytochemistry. DAPI signals indicate the total cell presence in the image (scale bars, 100 μm). (D) The expression of *OCT4*, *SOX2* and *NANOG*, which are human ESC-specific markers, was detected by RT-PCR (lane1, ALS patient-derived iPSCs; lane 2, normal-derived iPSCs; and lane 3, human fibroblast as a negative control). (E) The expression of *NESTIN*, *PAX6*, and *FOXG1* which are the specific markers for the human neural rosette, was detected by RT-PCR (lane 1, ALS patient-derived neural rosettes; lane 2, normal-derived neural rosettes; and lane 3, human fibroblast as a negative control). (F) Quantitative comparison of relative gene expressions between normal subjects and ALS patients by RT-qPCR in iPSCs. (G) Quantitative comparison of relative gene expressions between normal subjects and ALS patients by RT-qPCR in neural rosettes. NR, neural rosette, *P < 0.05, ^†^*P* < 0.1

The iPSCs from patient-derived fibroblasts were verified their pluripotency through AP staining (Fig 4A and 4B). Additionally, expression of OCT4 and SSEA4 through immunocytochemistry, and the expressions of OCT4, SOX2 and NANOG were confirmed in RNA level (Fig 4C and 4D). Neural rosettes from iPSCs were also confirmed in RNA level to verify their success of differentiation through the expressions of NESTIN, PAX6, and FOXG1 (Fig 4E). With these iPSCs and neural rosettes, we confirmed the patterns of gene expressions that are related to inflammation. This result in iPSCs was determined by RT-qPCR as follows: *CD14* (2.13-fold, *p* = 0.180), *IFNAR-1* (0.91-fold, *p* = 0.485), *TOLLIP* (1.56-fold, *p* = 0.002), *PIK3CA* (1.50-fold, *p* = 0.009), *IRAK4* (6.49-fold, *p* = 0.002), *MAPK1* (0.95-fold, *p* = 0.699), *MAPK9* (0.89-fold, *p* = 0.589), *IL-1β* (1.97-fold, *p* = 0.132), *IL-8* (8.38-fold, *p* = 0.002), *CXCL1* (3.5-fold, *p* = 0.002), and *CARD9* (1.27-fold, *p* = 0.394) (Fig 4F).

This result in neural rosettes was determined by RT-qPCR as follows: *CD14* (1.33-fold, *p* = 0.065), *IFNAR-1* (0.97-fold, *p* = 0.818), *TOLLIP* (1.50-fold, *p* = 0.065), *PIK3CA* (1.04-fold, *p* = 0.589), *IRAK4* (1.71-fold, *p* = 0.132), *MAPK1* (1.21-fold, *p* = 1.000), *MAPK9* (1.15-fold, *p* = 0.818), *IL-1β* (1.98-fold, *p* = 0.026), *IL-8* (1.14-fold, *p* = 0.589), *CXCL1* (1.79-fold, *p* = 0.026), and *CARD9* (1.30-fold, *p* = 0.065) (Fig 4G).

### Changes in TOLLIP expression according to progression of passages

**Fig 5:**
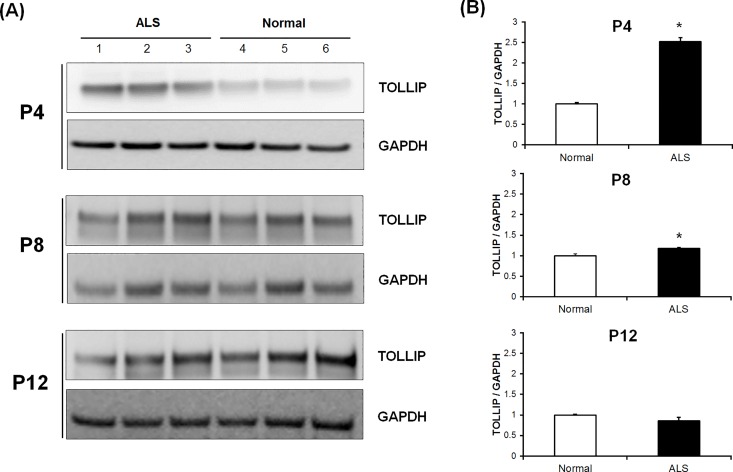
Validation of TOLLIP expression with progression of passages by western blotting. (A) Fibroblasts from normal subjects and patients with ALS were subcultured to the 12^th^ passage. Western blot analysis of protein extracted from the 4^th^, 8^th^, and 12^th^ passages (P4, P8, and P12, respectively) was performed using antibodies against TOLLIP and GAPDH (as a control). (B) With advancing passages, the expression levels of TOLLIP in patients with ALS were reduced compared to those in normal subjects. The numbers correspond to the ratio of the expression level of TOLLIP in patients with ALS compared to that in normal subjects. *P < 0.05

We did not expect to see overexpression of TOLLIP in fibroblasts of patients with ALS because it is known to have a modulatory role whereas the other five genes function as inducers of the inflammatory immune response. We hypothesized that expression of TOLLIP would change with the cellular aging process. To investigate whether expression of TOLLIP changes throughout the progression of disease, we performed western blot analysis of TOLLIP expression in fibroblasts of patients with ALS collected at passages 4, 8, and 12, respectively. At passages 4 and 8, TOLLIP was overexpressed in patients with ALS relative to controls (2.52-fold, *p*<0.001; 1.17-fold, *p* = 0.011); however, its expression decreased with increasing passages and the expression of TOLLIP in patients with ALS was lower than that in controls at passages 12 (0.85-fold; Fig 5).

### The expression of inflammation-related genes according to induced inflammation

**Fig 6:**
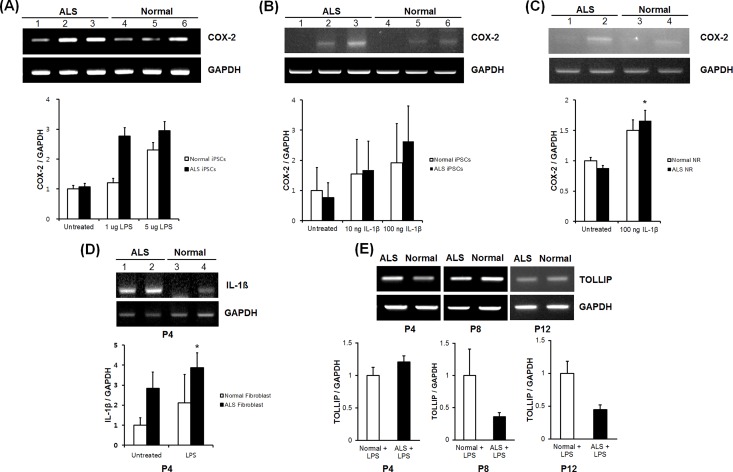
Expression of COX-2, IL-1β, and TOLLIP after treatment with lipopolysaccharide or IL-1β as an inflammatory stimulus. (A) Validation of COX-2 by induced inflammation with LPS in fibroblasts from ALS patients (lane 1, untreated ALS patient-derived iPSCs; lane 2, 1 μg/mL LPS-treated ALS patient-derived iPSCs; lane 3, 5 μg/mL LPS-treated ALS patient-derived iPSCs; lane 4, untreated normal-derived iPSCs; lane 5, 1 μg/mL LPS-treated normal-derived iPSCs; lane 6, 5 μg/mL LPS-treated normal-derived iPSCs). (B) Validation of COX-2 by induced inflammation with IL-1β in fibroblasts from ALS patients. Relative index graphs for comparison of COX-2 to GAPDH between ALS patients and normal controls (lane1, untreated ALS patient-derived iPSCs; lane 2, 10 ng /mL IL-1β-treated ALS patient-derived iPSCs; lane 3, 100 ng/mL IL-1β-treated ALS patient-derived iPSCs; lane 4, untreated normal-derived iPSCs; lane 5, 10 ng/mL IL-1β-treated normal-derived iPSCs; lane 6, 100 ng/mL IL-1β-treated normal-derived iPSCs). (C) Validation of COX-2 to compare in ALS patient- and normal control-derived neural rosettes (lane 1, untreated ALS patient-derived neural rosettes; lane 2, 100 ng/mL IL-1β-treated ALS patient-derived neural rosettes; lane 3, untreated normal-derived neural rosettes; lane 4, 100 ng/mL IL-1β-treated normal-derived iPSCs). (D) Validation of IL-1β by induced inflammation with LPS in fibroblasts from ALS patients (lane 1, untreated ALS fibroblasts; lane 2, 10 ng/mL LPS-treated ALS fibroblasts; lane 3, untreated normal fibroblasts; lane 4, 10 ng/mL LPS-treated normal fibroblasts) (E) Changes in TOLLIP expression according to progression of culture passages. TOLLIP expression in LPS-stimulated fibroblasts from patients with ALS decreased with increasing passage. *P < 0.05

During the study, we established iPSCs derived from normal controls and patients with ALS. After then, we differentiated established iPSCs into neural rosettes. When we confirmed expression of proinflammatory genes under the induction of inflammation with LPS or IL-1β, expressions of IL-1β or COX-2 by RT-PCR were upregulated in ALS patients compared with normal controls in iPSCs (Fig 6A and 6B) and neural rosettes (Fig 6C). First of all, we confirmed on iPSCs that COX-2 was expressed in a LPS dose-dependent manner. Moreover, while COX-2 was more expressed in the patient than normal, we could verify that the inflammation was more induced in ALS patient group than normal group (Fig 6A).

When another inflammatory inducing factor, IL-1β, was administrated, COX-2 was increased in a dose-dependent manner (Fig 6B). Additionally, on neural rosettes, when IL-1β was administrated, COX-2 increased its expression more in ALS patient group (Fig 6C). This result was similar with expression patterns in fibroblasts. Since the patterns of the proinflammatory gene expression according to induced inflammation in the three different types of cells—iPSCs, neural rosettes, and fibroblasts—were similar, these results showed that the phenomena were disease related. Consequently, the above results might give the proof that the gene expressions related to inflammation were upregulated in ALS via transcript screening on fibroblasts.

We then tested whether the inflammatory response induced by LPS stimulation leads to a change in TOLLIP expression in ALS according to progression of passages. The induction of an inflammatory response by LPS stimulation was confirmed by expression of the proinflammatory cytokine IL-1β using RT-PCR, and IL-1β was found to be overexpressed in fibroblasts from ALS patients compared with controls (Fig 6D). Expression of TOLLIP after LPS treatment was increased in fibroblasts from ALS patients at passages 4 but decreased with the progression of passages (Fig 6E).

## Discussion

From the confirmation of differentially expressed genes in normal- and patient-derived fibroblasts, this study demonstrated that the TLR and NLR signaling pathways are involved in ALS pathogenesis and identified TOLLIP, MAPK9, IL-1β, IL-8, and CXCL1. The iPSCs and neural rosettes were generated from the patient-derived fibroblasts, and these established cells showed the similar pattern with the fibroblast in the gene screening of TLR and NLR signaling pathways. In addition, we found that TOLLIP, a negative regulator of the TLR signaling pathway, was initially overexpressed, but its expression decreased with cellular aging.

TLRs recognize the specific patterns of pathogens and play a vital role in activating innate immunity. Recognition of pathogens by TLRs initiates signal transduction pathways, generating the expression of several genes such as those encoding proinflammatory cytokines (tumor necrosis factor-α [TNF-α], IL-1β, IL-6, and IL-12) and chemokines (IL-8, regulated on activation, normal T-cell expressed and secreted [RANTES], and macrophage inflammatory protein-1 [MIP-1], via the nuclear factor-kappaB [NF-κB] pathway), in addition to members of the MAPK pathway. Both innate immune responses and further development of antigen-specific acquired immunity are regulated by these gene products [27–29]. The previous study reported that CD14 and TLR2 were upregulated in animal models of various neurodegenerative diseases, including Alzheimer’s disease (AD), Parkinson’s disease (PD), and ALS [30]. These findings led researchers to focus on the TLR signaling pathway associated with innate immunity in ALS pathogenesis. Expression of mutant SOD1 in the mouse model of ALS facilitated the microglial neurotoxic inflammatory response through TLR2 [31]. Mutant SOD1 binds CD14, which is a co-receptor of TLR2 and TLR4. Microglial activation induced by mutant SOD1 was attenuated using CD14 blocking antibody or when the microglia lacked CD14 expression, suggesting that microglial activation via the CD14 and TLR pathways is a neuropathological hallmark of ALS [32, 33]. In addition, a previous study showed that CD14 and TLR2, potential indicators of the innate immune response, were upregulated in the spinal cords of mice and patients in ALS [30]. The results of this study supported that the TLR signaling pathway is associated with immune-related pathogenesis in ALS.

In addition to verifying the role of the TLR signaling pathway, this study yielded new observation that the NLR signaling pathway is involved in neuroinflammation in ALS. The NLR signaling pathway is thought to be a TLR-independent system for intracellular recognition of certain pathogens. NOD proteins recognize the core structures of bacterial peptidoglycans in the cytoplasm and have been implicated in the induction of NF-κB activity and the activation of caspases. These NOD proteins seem to function as cytosolic sensors for the induction of apoptosis and the induction and regulation of inflammatory responses [34]. NOD1 and NOD2 proteins undergo oligomerization upon recognizing the peptidoglycan motif, resulting in activation of the MAPK pathway and the transcription factor NF-κB [34, 35]. Thus, the NLR signaling pathway induces transcription of proinflammatory cytokines -IL-1β, IL-18, TNF-α, IL-6- and chemokines such as CXCL1.

The proinflammatory cytokines IL-1β and IL-8 are induced by the TLR and NLR signaling pathways. IL-1β is a vital mediator of the inflammatory response and is implied in a variety of cellular phenomenon, including cell proliferation, differentiation, and apoptosis. Pathologic activation of glia in ALS has been widely characterized and is marked by enhanced production of potentially cytotoxic molecules such as reactive oxygen species, inflammatory mediators such as COX-2, and proinflammatory cytokines such as IL-1β, TNF-α, and IL-6 [2]. In ALS, mutant SOD1 activates caspase-1 and IL-1β in microglia. Mutant SOD1-induced IL-1β correlates with amyloid-like misfolding and is independent of dismutase activity, suggesting that IL-1β affects significantly to disease progression in the mouse model of ALS by promoting neuroinflammation [36]. However, this result is controversial. Because mice with lacking IL-1β show unchanged disease onset, the result suggests that inflammation is not a starting factor in the disease [37]. IL-8, a chemokine produced by macrophages and other cells, is an essential mediator of the immune response in the innate immune system. IL-8 can be secreted by any cell type possessing the TLR that is involved in the innate immune response. This cytokine primarily targets the neutrophil granulocytes and is often associated with inflammation. In addition, IL-8 levels are known to increase under oxidative stress leading to recruitment of inflammatory cells and a further increase in oxidative stress mediators, making IL-8 a key factor in localized inflammation [38]. A previous investigation reported that an increased level of IL-8 in the cerebrospinal fluid (CSF) of patients with ALS suggests stimulation of a proinflammatory cytokine cascade after microglial activation [39]. Elevated levels of IL-8 in CSF from patients with ALS showed a negative correlation with ALSFRS-R score and alterations in chemokines were presumed to correlate with the clinical course of ALS [40]. Our data also showed upregulation of IL-1β in fibroblasts from ALS patients; however, it is not clear whether upregulation of IL-1β is the result or the cause of activation of TLR and NLR signaling. IL-8 has been reported to be a potent inducer of the CXCR1, CXCR2, and the chemokine CXCL1 [41]. CXCL1 has been reported to be overexpressed in gastric, colon, and skin cancers [42, 43] and is known to recruit oligodendrocytes in multiple sclerosis when coupled with CXCR2 [44]. CXCL1 was overexpressed in ALS fibroblasts in our study; however, its role in ALS pathogenesis is not clear.

MAPK9 was reported to be associated with mitochondrial dysfunction and glucocorticoid receptor signaling in neurodegenerative disorders using pathway analysis [45]. Pathway analysis in ALS using a genome-wide association study identified the MAPK signaling pathway as the one of the top candidate pathways [46]. In addition, aberrant expression and activation of p38 MAPK in motor neurons and microglia may play a role in the development and progression of ALS [47]. In a recent study [48], TAR DNA-binding protein 43 (TDP-43) depletion in microglia was shown to upregulate COX-2 expression and prostaglandin E2 (PGE2) production through activation of MAPK/ERK signaling. MAPK9 and CXCL1 have not been clearly associated with ALS, and there are no studies on their relationship to ALS. Upregulation of these two genes provides new clues for the better understanding of the mechanism of ALS.

TOLLIP, a member of the TLR signaling pathway, acts as a negative regulator of the TLR signaling pathway. TOLLIP associates with IL-1R and TLR4 after LPS activation, inhibiting the immune response mediated by TLR. In addition, TOLLIP binds directly to IRAK-1 and prevents NF-κB activation by inhibiting autophosphorylation of IRAK-1 [49–51]. Both NF-κB and MAPK pathways can be activated when endogenous IRAK-4 is overexpressed and interacts with IRAK-1 and TRAF6 in an IL-1-dependent manner [52]. Overexpression of TOLLIP leads to inhibition of the TLR signaling pathway. However, TOLLIP does not seem to play a neuroprotective role. When stimulated by IL-1β and LPS, TOLLIP-deficient mice showed normal activation of NF-κB and the MAPK signaling pathway, but significantly reduced production of proinflammatory cytokines. Therefore, TOLLIP is considered as a modulatory role, controlling the induction of inflammatory cytokines [53]. A previous study analyzing brain gene expression profiles of immune-related genes in aging and Alzheimer’s disease (AD) showed that TOLLIP was downregulated in the brains of aged subjects and AD compared to the brains of young subjects, whereas genes reflecting activation of microglia such as CD14, TLR2, and TLR4 were upregulated in the brains of aged subjects and AD. The researchers concluded that downregulation of TOLLIP attenuates TLR signaling and microglial activation [54]. In the previous study, the expression of TOLLIP was highest in the brains of young subjects, followed by the aged brain, and was lowest in the brains of subjects with AD. We carried out additional experiments for a better understanding of why TOLLIP was upregulated in passages 4 of ALS fibroblasts in our studies. Based on results from the research mentioned above, we suggested that the expression of TOLLIP might change temporally according to the cellular aging process, and found that expression of TOLLIP thus decreased with aging of ALS cells. The similar results were further confirmed with LPS treatment for stimulation of inflammation. We postulate that overexpression of TOLLIP in the early stages of passages represents the compensatory activity of the cells, and that expression of TOLLIP decreases with additional passages because of loss of compensation. A previous study reported that microglia and inflammatory cells are highly dynamic over the course of the disease and time-dependent modification is important for the development of ALS [55]. Expression profiles of cytokines such as TNF-α, transforming growth factor-β1 (TGF-β1), and macrophage colony-stimulating factor (M-CSF) in the spinal cord of the mouse model of ALS showed upregulation according to aging, suggesting that this temporal profile of expression might contribute to the disease phenotype [56]. In this study, we confirmed the low proliferation rate and advanced senescence of fibroblasts in ALS according to the progression of cellular passages. This cellular aging process may contribute to change TOLLIP expression according to the passages. We suggest that TOLLIP expression reflects the temporally controlled neuroprotective and neurotoxic presentation of ALS.

RNA for gene expression profiling was isolated from the fibroblasts of subjects. The hallmark of ALS pathogenesis is motor neuronal death in the spinal cord and motor cortex, whereas fibroblasts are non-neuronal cells. Although investigation of neuronal cells might provide more information on the pathogenesis of ALS, biopsy of neuronal tissues is invasive and not an easy technique. In contrast, fibroblasts are gladly accessible and may hold potential for short-term, rapid diagnostic and prognostic plans. Several studies have examined pathologic features in fibroblasts from ALS patients [57–59]. They showed that fibroblasts recapitulate some of the hallmark abnormalities in TDP-43 observed in neuronal cells [57] and reported findings of increased membrane potential and decreased mitochondrial content in ALS fibroblasts [58]. Additionally, gene expression profiling of fibroblasts from ALS demonstrated differential expression of genes related in RNA processing and the stress response, a noteworthy decrease in miRNA production, and a reduced response to hypoxia, suggesting that fibroblasts can act as cellular models for ALS [59].

Since ALS is a CNS disease, study of gene expressions related to inflammation in fibroblasts directly could not represent the inflammatory pathway in ALS, and it would be the best if we could obtain spinal fluid or post mortem tissues from patients. Nevertheless, the simple way to get patients’ samples is primary culture of fibroblasts via skin biopsy. Therefore, we have strived to established neural lineage cells instead of getting the samples of spinal fluid or post mortem tissues. We have established iPSCs derived from normal and patients’ fibroblasts and generated neural rosettes from the iPSCs. The study of cells that differentiated into neural lineage cells could be an alternative proposal. In this study, we studied the certain inflammation-related genes in three different types of cells such as fibroblasts, iPSCs, and neural rosettes, and confirmed the genes that considered as representing the certain inflammatory pathways.

Taken together, besides other studies, this study showed the results in three different types of cells to overcome the limitation of small sample size and the type of the original sample. Since the original sample was the patient-derived fibroblasts and there were only three patients, these were considered to be the limitation for the CNS disease research. However, while this study established iPSCs and neural rosettes from the fibroblasts, these three different types of cell lines added more various on sample research, and showed CNS related result. Nonetheless, the results of our study should be confirmed by gene expression profiling of motor neuron cells in the future.

## Conclusions

Inflammatory responses might play a vital role in the pathogenesis of motor neuron damage in ALS. Gene expression profiling and pathway analysis showed that the TLR and NLR signaling pathways are related in the pathogenesis of ALS through fibroblasts, iPSCs and neural rosettes. These pathways are related to pathological innate immunity and neuroinflammation. Overexpression of genes related to inflammation, such as *TOLLIP*, *MAPK9*, *IL-1β*, *IL-8*, and *CXCL1*, was validated and changes in TOLLIP expression associated with cellular aging were observed. A future research will be focused on the possible contribution factors that related to the etiology of ALS, so might be identified the therapeutic targets.

## Supporting Information

S1 Fig Sequence analysis of C9ORF7 gene. (A) The sequencing result of C9ORF7 in ALS patient number 1 (B) The sequencing result of C9ORF7 in ALS patient number 2 (C) The sequencing result of C9ORF7 in ALS patient number 3
(PDF)

S1 Table Raw data for 17,025 differentially expressed transcripts. Raw data of RNA sequencing to identify genes differentially expressed between ALS patients and controls; 17,025 differentially expressed transcripts were obtained.
(XLSX)

S2 Table Raw data for 1215 differentially expressed transcripts. Among the total of 17,025 differentially expressed transcripts, 1215 transcripts were showing 2.0-fold up- or down-regulation. The expression levels of 626 transcripts were downregulated and those of 589 transcripts were upregulated in preeclampsia patients compared to controls.
(XLSX)
