# Supplementary figures and images for: Elucidation of Relevant Neuroinflammation Mechanisms Using Gene Expression Profiling in Patients with Amyotrophic Lateral Sclerosis
Source: PLoS One. 2016 Nov 3;11(11):e0165290. doi: 10.1371/journal.pone.0165290 (PMC5094695; doi:10.1371/journal.pone.0165290)

**(A)**

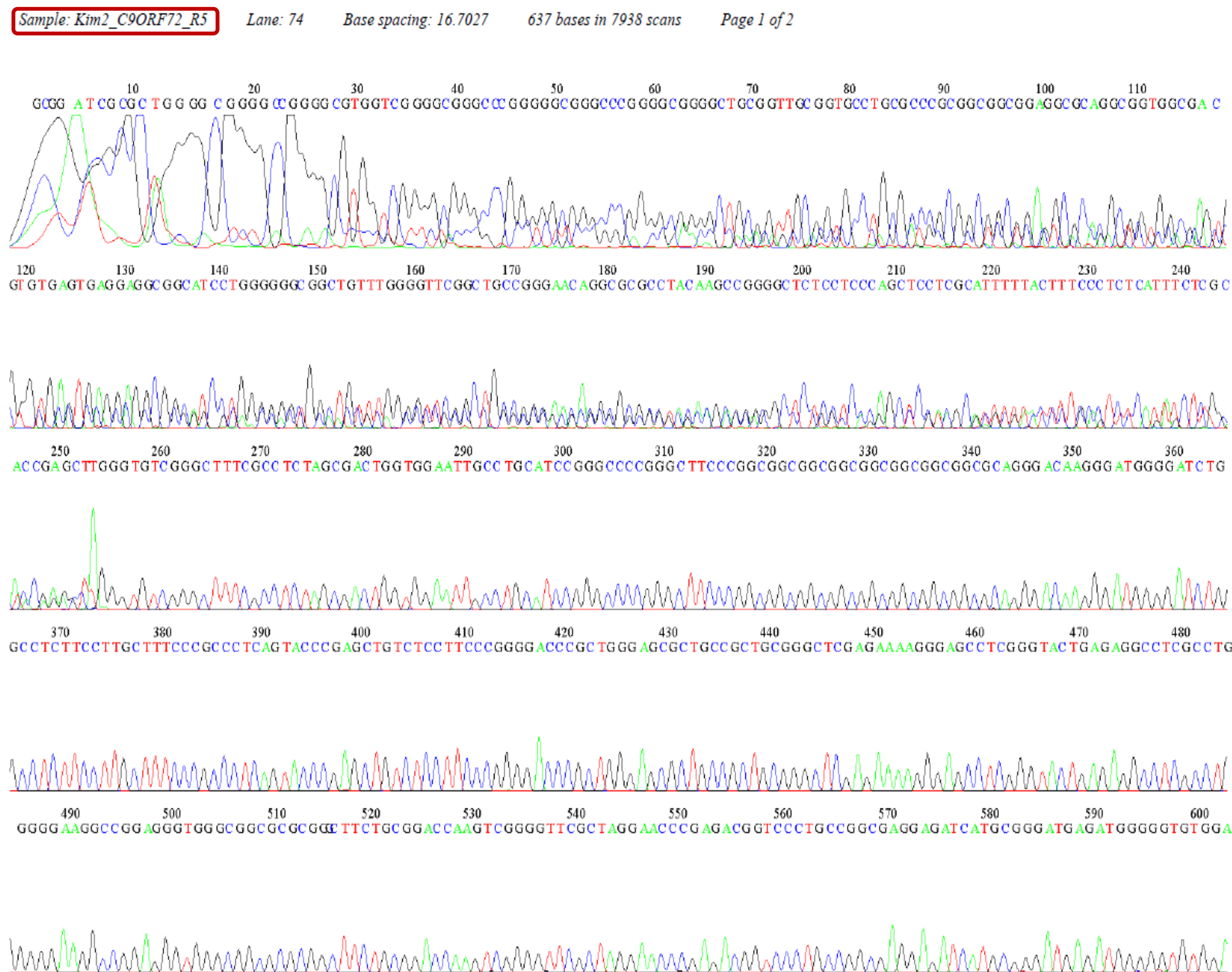

**(C)**

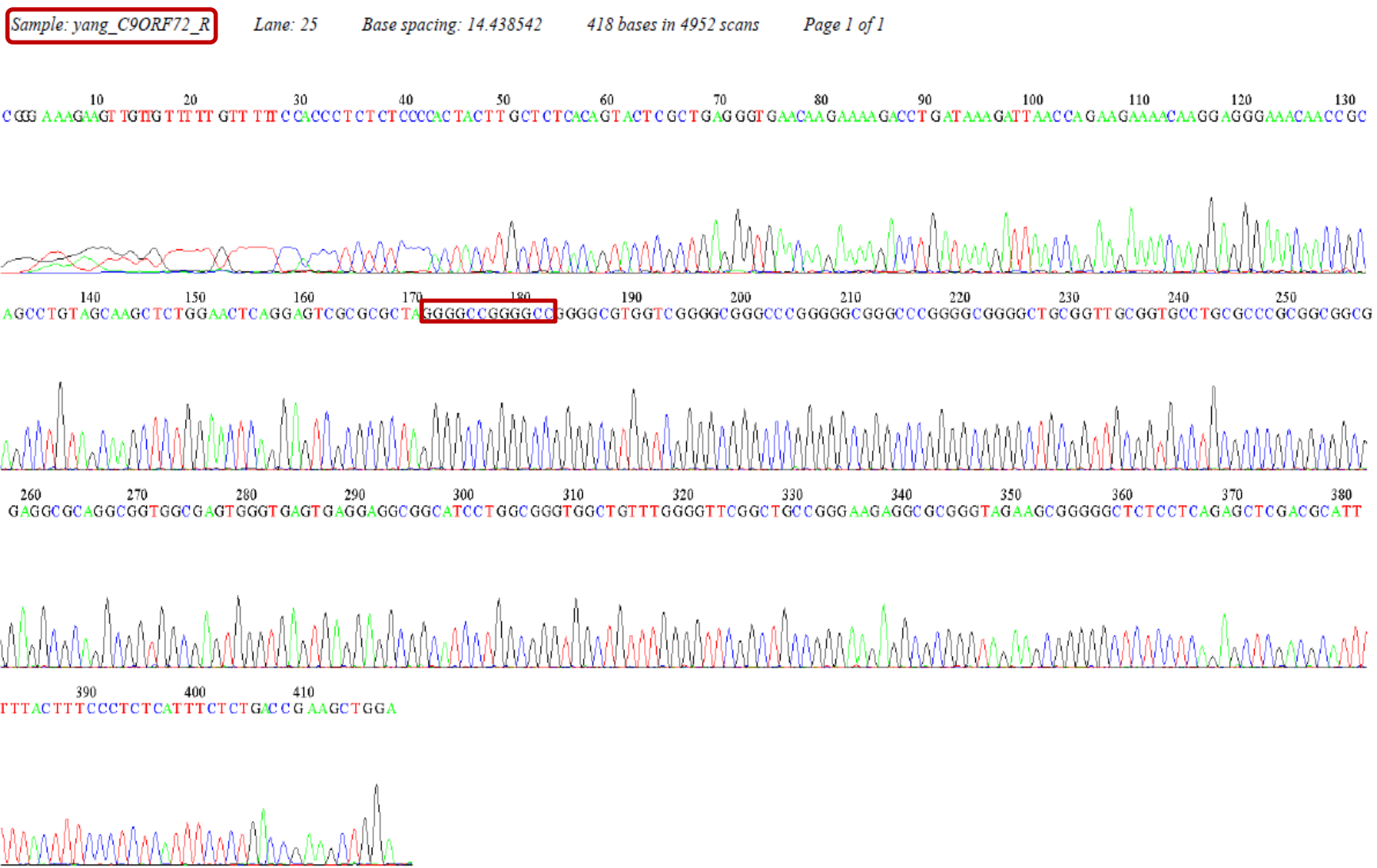

**(B)**

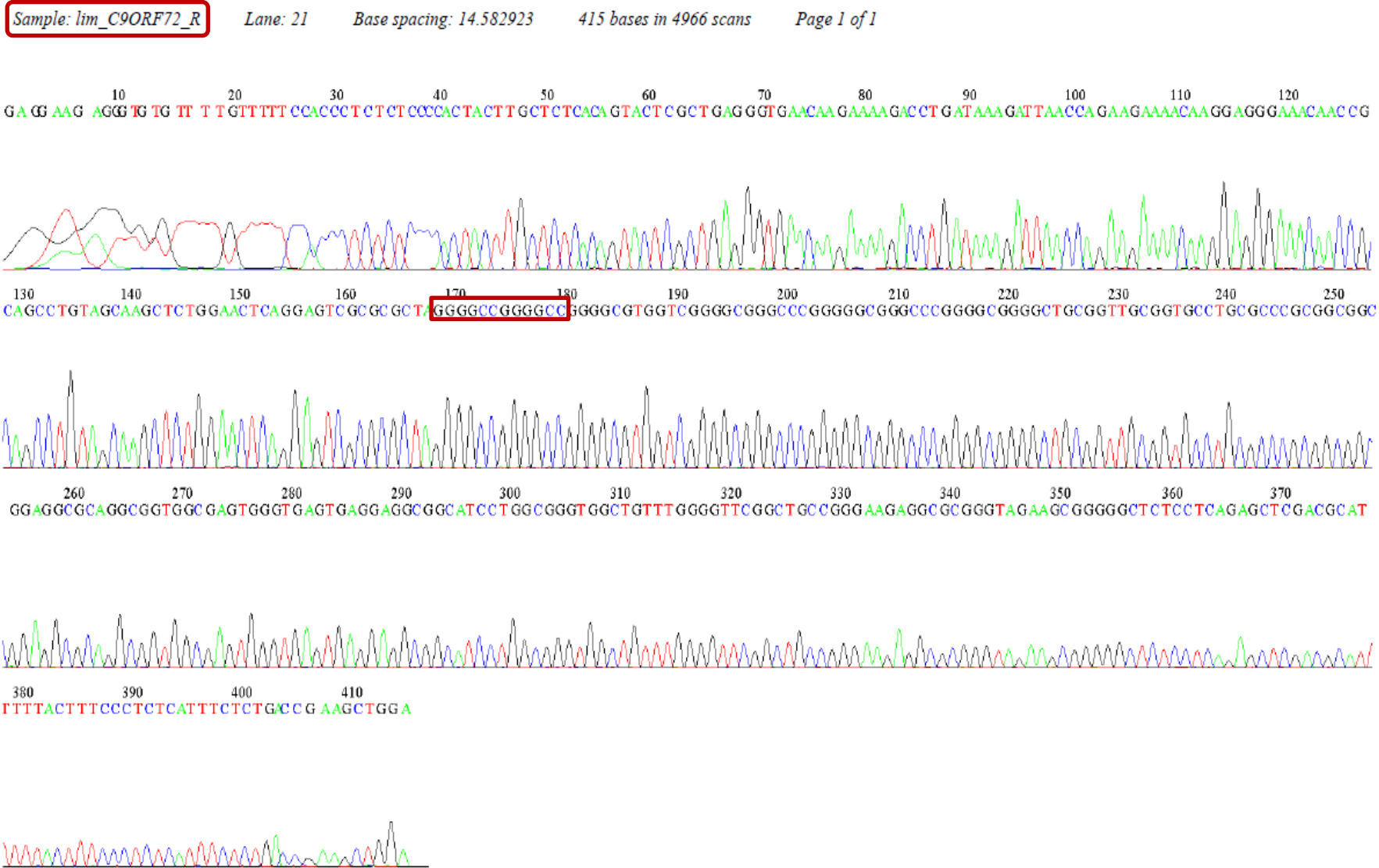

Supplement: S1 Fig — (A) The sequencing result of C9ORF7 in ALS patient number 1 (B) The sequencing result of C9ORF7 in ALS patient number 2 (C) The sequencing result of C9ORF7 in ALS patient number 3 (PDF) [file pone.0165290.s001.pdf]
